# Coupled one-off alternate furrow irrigation with nitrogen topdressing at jointing optimizes soil nitrate-N distribution and wheat nitrogen productivity in dryland

**DOI:** 10.3389/fpls.2024.1372385

**Published:** 2024-05-28

**Authors:** Ming Huang, Wenna Li, Chuan Hu, Jinzhi Wu, Hezheng Wang, Guozhan Fu, Muhammad Shaaban, Youjun Li, Guoqiang Li

**Affiliations:** ^1^ College of Agriculture, Henan University of Science and Technology, Luoyang, China; ^2^ Key Laboratory of Huang-Huai-Hai Smart Agricultural Technology, Ministry of Agriculture and Rural Affairs, Zhengzhou, China; ^3^ Institute of Agricultural Information Technology, Henan Academy of Agricultural Sciences, Zhengzhou, China

**Keywords:** one-off alternate furrow irrigation, topdressing N, dryland winter wheat, soil nitrate-N, grain yield, N use efficiency

## Abstract

The judicious management of water and nitrogen (N) is pivotal for augmenting crop productivity and N use efficiency, while also mitigating environmental concerns. With the advent of the High−Farmland Construction Program in China, one−off irrigation has become feasible for most dryland fields, presenting a novel opportunity to explore the synergistic strategies of water and N management. This study delves into the impact of one−off alternate furrow irrigation (AFI) and topdressing N fertilizer (TN) on soil nitrate−N distribution, and N productivity—including plant N accumulation, translocation, and allocation, and grain yield, protein content, N use efficiency of winter wheat (Triticum aestivum L.) in 2018−2019 and 2019−2020. Experimental treatments administered at the jointing stage comprised of two irrigation methods—every (EFI) and alternative (AFI) furrow irrigation at 75 mm, and two topdressing N rates—0 (NTN) and 60 (TN) kg N ha^−1^. Additionally, a conventional local farmer practice featuring no irrigation and no topdressing N (NINTN) was served as control. Compared to NINTN, EFINTN substantially increased aboveground N accumulation, grain yield, and protein yield, albeit with a reduction in grain protein content by 8.1%−10.6%. AFI, in turn, led to higher nitrate−N accumulation in the 60−160 cm soil depth at booting and anthesis, but diminished levels at maturity, resulting in a significant surge in N accumulation from anthesis to maturity and its contribution to grain, N fertilizer partial factor productivity (PFPN), and N uptake efficiency (NUPE), thereby promoting grain yield by 9.9% and preserving grain protein content. Likewise, TN enhanced soil nitrate−N at key growth stages, reflected in marked improvements in N accumulation both from booting to anthesis and from anthesis to maturity, as well as in grain yield, protein content, and protein yield. The combination of AFI and TN (AFITN) yielded the highest grain yield, protein content, with PFPN, NUPE, and N internal efficiency outstripping those of EFINTN, but not AFINTN. In essence, one−off AFI coupled with TN at the jointing stage is a promising strategy for optimizing soil nitrate−N and enhancing wheat N productivity in dryland where one−off irrigation is assured.

## 1 Introduction

Wheat stands as the cornerstone of nutrition and vegetable protein in the human diet, occupying over 20% of the world’s arable territory and feeds about 30% of the world population (Zhang et al., 2019; Liu et al., 2020). Despite this, around 75% of wheat production emanates from drylands area in world—regions mainly characterized by aridity, semi−aridity, and semi−humid drought−prone (Khan et al., 2017; Zhang et al., 2019). Water scarcity and nutrient shortfalls impose significant constraints on the pursuit of enhanced and consistent wheat yields in these regions (Luo et al., 2021). Soil moisture deficits not only curtail nutrient uptake efficiency but also compromise the productivity of both water and nutrients, leading to low and unstable wheat yields (Gan et al., 2013; Zhang et al., 2020). Nonetheless, the swift progression of the High−Standard Farmland Construction Program in China, culminating in 66.7 million hectares of high−caliber farmland by 2022 and plans for an additional 13.3 million hectares during the 14^th^ Five−Year Plan, has cemented the provision of one−off irrigation for wheat growth across numerous dryland areas—locales previously bereft of irrigation (Wang et al., 2022; Zhao et al., 2023b). However, management strategies that leverage this one−off irrigation are still underdeveloped, it is a urgent necessity to refine water and nutrient management tactics to grasp this one-off irrigation opportunity to bolster wheat productivity.

Nitrogen (N), a linchpin for crop physiological and agronomic health, is crucial for robust plant growth and development (Wang et al., 2014). An adequate water regimen enhances grain N uptake and overall crop productivity (Kumari et al., 2017); conversely, water imbalances can precipitate N inefficiencies (Sarker et al., 2020; Zhao et al., 2023b). Research by Jia et al. (2021) acknowledged that alternate furrow irrigation (AFI) targets the root zone with precision, thereby optimizing water provisioning, bolstering shoot N accumulation, and augmenting N use efficiency (NUE), ultimately yielding richer wheat harvests. However, instances under low rainfall condition have shown AFI to be detrimental to N uptake, leading to decreased crop yields when contrasted with conventional every furrow irrigation (EFI) due to diminished irrigation volumes (Benjamin et al., 1997; Sepaskhah and Hosseini, 2008). Yet, combining AFI with other approaches, such as N management and planting modes, has been demonstrated to enhance NUE in wheat crops (Ghasemi-Aghbolaghi and Sepaskhah, 2018). Topdressing nitrogen (TN), a strategic intervention for crop yield improvement, furnishes N nutrition vital for subsequent growth stages, particularly when soil N content is suboptimal (Li et al., 2020; Ji et al., 2021; Fu et al., 2022). Zain et al. (2021) discovered that TN applied at the jointing stage syncs with wheat’s N demands, optimizing NUE in China North Plain. In drought−prone zones, a one-off irrigation based on 0−40 cm soil moisture levels at the regreening stage and coupled with a 50% N fertilizer topdressing, has proven to increase shoot N accumulation, NUE, and grain yield while minimizing soil nitrate-N residue at harvest (Zhao et al., 2023b). These insights suggest that judicious irrigation paired with TN at the proper stage are pivotal for elevating grain yield and NUE, enabling an environmentally congenial wheat production system. However, farmers usually apply all N fertilizers before sowing due to irrigation and labor constraints, which lead to a misalignment between the wheat’s water and N requirements and result in some environmental issues (Wang and Li, 2019). Thus, this condition reinforce the need for AFI and TN protocols in enhancing winter wheat productivity in drylands.

The presence of nitrate−N in soil is integral for plant N uptake; however, if it exceeds safety thresholds, it is at risk of leaching, denitrification, and emissions in winter wheat production systems (Zhou and Buterbach-Bahl, 2014; Dai et al., 2015; Huang et al., 2017; Zhao et al., 2023a). Notably, dryland farms in China often exhibit substantial soil nitrate−N concentrations, such as 601 kg ha^−1^ in 100−180 cm (Dai et al., 2015), 1065 kg ha^−1^ in 0−300 cm (Guo et al., 2010), and a staggering 708−1500 kg ha^−1^ within a 0−380 cm soil profile (Zhao et al., 2023a)—figures that underscore the urgency for agro−operations focused on optimizing soil nitrate−N to favor crop uptake and minimize environmental risks (Adel et al., 2019; Wang and Li, 2019).

The furrow-seeded (FS) framework, is widely applied in drylands for the purpose to increase crop yields and N use efficiency (Sharma et al., 2012; Gan et al., 2013; Mehdi et al., 2017; Ali et al., 2018; Zhang et al., 2020; Yang et al., 2021). In FS system, the ridges in the field alternate with the corresponding furrows, which made irrigation more conveniently and precisely (Jia et al., 2021). Previous studies have demonstrated that, under RF, the winter wheat yield in dryland could be significantly increased via irrigation (Ali et al., 2018; Li et al., 2019). For example, Li et al. (2019) found that a total amount of irrigation of 165 mm during the growth stage increased wheat yield by 46.1%. Luo et al. (2020) reported that the irrigation of 7.8–11.8 mm at wintering and jointing increased wheat yield by 10.0–27.1%. However, these researches mainly focus on optimizing amount and/or its technology of irrigation, but not pay attention in one-off irrigation. Moreover, the influence of coupled FI and TN techniques on soil nitrate−N and N productivity in FS winter wheat remains elusive. This gap inhibits the full utilization of water resources, which come from High−Standard Farmland Construction efforts, to boost wheat productivity.

Within this context, our study proposed that coupled AFI with TN could optimize soil nitrate−N and maximize N productivity in winter wheat. Employing AFI and TN at the jointing stage of winter wheat in a semi−humid and drought−prone region, this research aims to: (1) elucidate the effects of FI, AFI, and TN, as well as their interactions, on soil nitrate−N accumulation; (2) evaluate their impact on aboveground N accumulation, translocation, allocation, grain yield, grain protein content, protein yield, and N use efficiency; and (3) identify an optimal agronomic strategy that synergizes soil nitrate−N, crop yield, quality, and efficiency within the FS system in drylands where one−off irrigation is assured.

## 2 Materials and methods

### 2.1 Study site description

From October 2018 and June 2020, a two−year field experiment was carried out at Nandasu village, located in Xiaolangdi town, Mengjin district, Luoyang, Henan province—one of typical dryland winter wheat producing regions in China. The study site was characterized by an average annual air temperature of 13.7°C and a mean annual frost−free period of 210 days, the area enjoys an average annual sunshine duration of 2196 hours and receives an average annual precipitation of 650 mm. Notably, around 60% of this rainfall occurs from June to September, which marginally overlaps with the winter wheat year. The predominant cropping system in this locale is the winter wheat−summer maize rotation, with winter wheat typically sown in early to mid−October and harvested at the beginning of June the following year.

**Figure 1:**
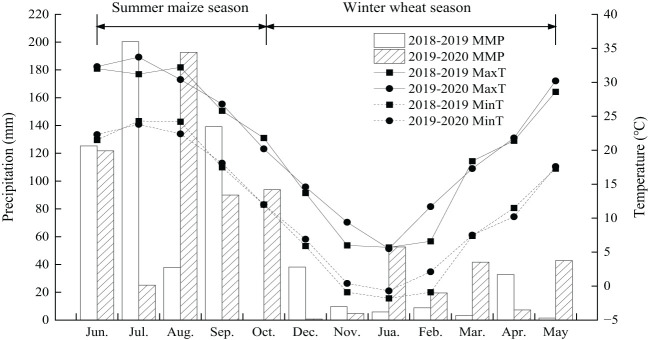
Monthly precipitation at the experimental site from June 2018 to May 2020.

During the two experimental years, the recorded annual precipitation was 602.1 mm in 2018−2019 and 692.7 mm in 2019−2020, with 16.5% and 38.0% of this, respectively, occurring during the winter wheat season (**Figure 1**). The experimental site’s soil, formed from cinnamon−colored parent material and classified as a calcareous Eum−Orthic Anthrosol according to Chinese soil taxonomy, displayed consistent baseline properties at the initiation of the experiment for both years. Within the upper 0−20 cm soil layer, assessments revealed a soil field capacity ranging from 27.3% to 27.4%, bulk density between 1.35 and 1.36 g cm^-3^, pH of 8.2, organic matter content averaging 13.1−13.2 g kg^−1^, total N content fixed at 0.81 g kg^−1^, available phosphorus contents ranging from 12.1 to 13.2 mg kg^−1^, and available potassium levels between 121.6 and 125.4 mg kg^−1^.

### 2.2 Experimental design and field management

Our study incorporated a two−factor experimental design beyond the control treatment, focusing on furrow irrigation (FI) techniques and topdressing nitrogen (TN) rates. The primary treatments involved two FI techniques: every furrow irrigation (EFI) and alternate furrow irrigation (AFI), each with 75 mm of water applied at the jointing stage. For the secondary treatments, we employed two TN rates: a Zero−N topdressing (NTN) at 0 kg ha^−1^, and a N topdressing (TN) at 60 kg ha^−1^, administered concurrently with the irrigation. Additionally, the traditional local farmer planting practice featuring no irrigation and no topdressing N (NINTN) was served as control. Consequently, the experimental framework encompassed five distinct treatments:

1. Conventional no irrigation and no topdressing N (NINTN).

2. Every furrow irrigation without topdressing N (EFINTN).

3. Alternate furrow irrigation without topdressing N (AFINTN).

4. Every furrow irrigation with topdressing N (EFITN).

5. Alternate furrow irrigation with topdressing N (AFITN).

The application amount of basal fertilizer is set according to local farmer’ practice, and both the application amount of irrigation and N fertilizer topdressing are recommended by local agricultural experts. The specific amounts for irrigation and fertilizer application are detailed in **Table 1**.

**Table 1:** The irrigation amount and fertilizer application rates in different treatments in 2018–2019 and 2019–2020.

Treatments	Irrigation (mm)	Fertilizer application rates(kg ha^−1^)			
		Basal N	Basal P_2_O_5_	Basal K_2_O	Topdressing N
NINTN	0	172.5	75	45	0
EFINTN	75	172.5	75	45	0
AFINTN	75	172.5	75	45	0
EFITN	75	172.5	75	45	60
AFITN	75	172.5	75	45	60

NINTN, no irrigation with no topdressing N; EFINTN, every furrow irrigation with no topdressing N; AFINTN, alternate furrow irrigation with no topdressing N; EFITN, every furrow irrigation with topdressing N; AFITN, alternate furrow irrigation with topdressing N.

Our study utilized a completely randomized plot design with three replications for each treatment. Each plot measured 20 meters in length and 6.12 meters in width, and all were situated within the same field with a 1−meter buffer between each. Upon sowing, we utilized a no−till fertilizer seeder (model 2BMQF−6/12A, produced by Luoyang Xinle Machinery Co., Ltd) to apply compound fertilizers (N:P_2_O_5_:K_2_O ratio of 23:10:6) at a rate of 750 kg per hectare as the base fertilizer.

**Figure 2:**
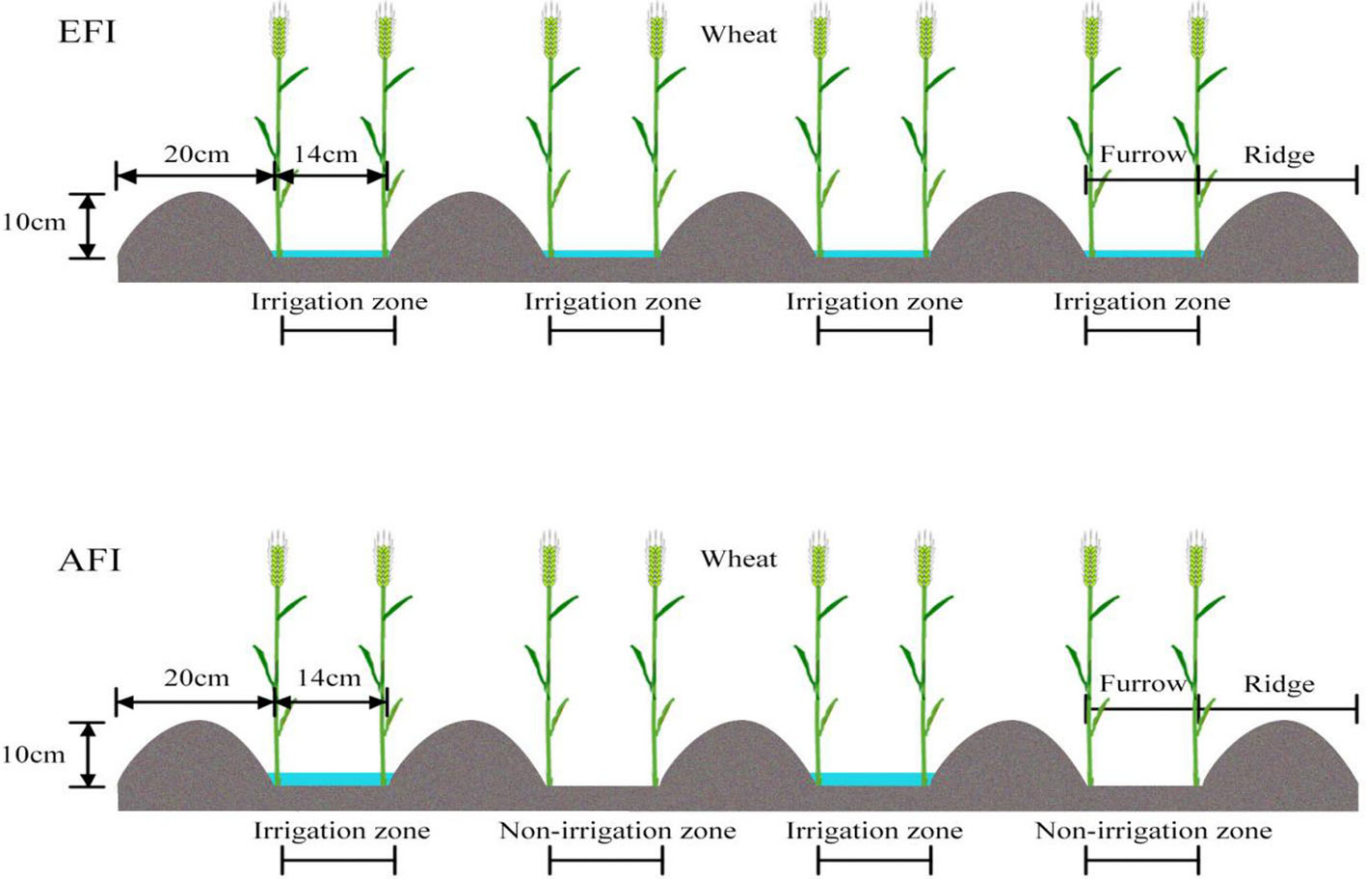
A schematic diagram of EFI and AFI system.

The 2BMQF−6/12A no−till seeder is designed to perform multiple tasks—including furrowing, ridging, fertilizing, sowing, and soil repacking—simultaneously. Post−sowing, the equipment was adjusted to create ridges 20 cm wide and 10 cm high, with furrows that were 14 cm wide. Consequently, the spacing for wheat plants in the wider rows was 20 cm, compared to 14 cm in the narrower rows, averaging to 17 cm overall (**Figure 2**).

Winter wheat variety Zhoumai36 was sown using a seeding rate of 187.5 kg per hectare, at a depth of 3−5 cm within the furrows. The base fertilizers were precisely drilled between two seed rows at a depth of 10 cm. Sowing dates were October 13, 2018, and October 15, 2019, with respective harvest dates of May 30, 2019, and June 2, 2020. The previous crop in the both two years were summer maize (Zhengdan958), which harvest at early-October. Irrigation conducted at the jointing stage (Zadoks 31) on March 19, 2019, and March 22, 2020, with both the AFI and EFI treatments receiving 75 mm, calibrated for the entire plot area and regulated using a mechanical water meter (with 0.01m³ accuracy and operating at outlet valve pressures between 0.10−0.12 MPa). Specifically, each plot received 9.18 m³ of water, and the irrigation space per furrow was 2.8 m² (20 m × 0.14 m). Under EFI treatment, the total irrigated area per plot was 50.4 m² (20 m × 2.52 m), with 0.51 m³ of water for each irrigated furrow. Conversely, under AFI treatment, the irrigated area per plot was halved to 25.2 m² (20 m × 1.26 m), with 1.02 m³ of water for each irrigated furrow—effectively doubling the irrigation volume per furrow compared to EFI (**Figure 2**).

For TN treatments, a uniform rate of 60 kg N per hectare was hand−broadcast in the watering furrows just before the irrigation event. To manage weeds, pests, and diseases, we employed the same herbicides and pesticides used by local farmers.

### 2.3 Measurements and methods

#### 2.3.1 Soil nitrate−N

During the 2018−2019 and 2019−2020 years, soil core samples were collected randomly at key phenological stages: booting (Zadoks 43), anthesis (Zadoks 65), and maturity (Zadoks 94). We sampled these using a soil auger with an internal diameter of 4.0 cm, extracting cores from 0 to 200 cm soil depths in 20 cm intervals. For NINTN and EFI treatments, we randomly took three soil cores from the center between two plant rows within a furrow. In the case of AFI treatments, we collected three soil cores from the center of irrigated furrows and another three from the center of non−irrigated furrows. The individual soil samples from identical depths and the same plot were combined, yielding round 300 grams after a homogeneous mix. This composite sample was immediately sealed in a labeled plastic bag to preserve it for further laboratory analysis. In the laboratory, the nitrate-N was quantified with the method described by Huang et al. (2017), fresh soil samples weighing 5.0 g were extracted with 50 mL of a 1.0 mol L^-1^ KCl after shaking it continuously for 1h. We then filtered the resultant mixture and promptly measured the nitrate−N concentration within the filtrate using a high−resolution digital colorimeter, the AutoAnalyzer 3 (AA3) from SEAL Company in Germany.

The soil nitrate−N accumulation (SNA, kg N ha^–1^) in the 0−200 cm soil profile was calculated as follows (Dai et al., 2015; Huang et al., 2017):


(1) SNA=Ti×Di×Ci×0.1

where T_i_ is the soil layer thickness (cm), D_i_ is the soil bulk density (g cm^−3^), C_i_ is the soil nitrate concentration (mg kg^−1^), i.e., i = 20, 40, 60, 80, 100, 120, 140, 160, 180, 200. The soil bulk density of 1.32, 1.34 and 1.38 g cm^−3^ was used in 0−20, 20−40, and 40−200 cm according to the average value of local field, and 0.1 is the conversion coefficient.

#### 2.3.2 Plant N

At the jointing (Zadoks 31) stage, we collected four 0.5 m−long samples of winter wheat from random locations within the experimental field. Additional samples were harvested from each plot at anthesis (Zadoks 65) and maturity (Zadoks 94) stages. The process involved counting the tillers and removing the roots using scissors where the stem intersected with the root system. The aboveground biomass at both anthesis and maturity stages was categorized into stem, leaves, sheath, and ear components. Specifically, at maturity, the ear was further divided into grain and chaff (glume + rachis). Subsequently, the biomass was oven−dried at 105°C for 30 minutes, followed by drying at 65°C for 24 hours to establish the dry weight. The oven−dried samples, encompassing grain, straw, and glume, were finely ground in preparation for chemical analysis. Utilizing a mixture of sulfuric acid and hydrogen peroxide (H_2_SO_4_−H_2_O_2_), the samples were digested, allowing us to determine N concentrations in the digestion solution with an AutoAnalyzer 3 (AA3, SEAL Company, Germany) using the method prescribed by Huang et al. (2017). The N accumulation in each plant organ was calculated by multiplying the organ’s dry weight (expressed in kg per hectare) by its respective total N concentration (in g kg^−1^). The total aboveground N accumulation (NA, in kg ha^−1^) was then computed by aggregating the N accumulation figures for each organ. Further, we calculated various N−related parameters including accumulation, translocation, and allocation based on the Equations 2-7 (Huang et al. 2021).


(2) $$\begin{array}{c} \text{Aboveground N accumulation from jointing to anthesis}\\ \left({\text{NA}}_{\text{JTA}},{\text{kg ha}}^{-1}\right)\\ =\text{Aboveground N accumulation at anthesis}\\ -\text{Aboveground N accumulation at jointing} \end{array}$$

(3) $$\begin{array}{c} \text{Aboveground N accumulation from anthesis to maturity}\\ \left({\text{NA}}_{\text{ATM}},{\text{kg ha}}^{-1}\right)\\ \;=\text{ Aboveground N accumulation at maturity}\\ –\text{Aboveground N accumulation at anthesis} \end{array}$$

(3) $$\begin{array}{c} \text{Rate of periodical N accumulation to total N accumulation}\left(\%\right)\\ =\text{Periodical aboveground N accumulation}\\ ÷\text{Aboveground N accumulation at maturity} \end{array}$$

(4) $$\begin{array}{c} \text{Pre-anthesis N translocation amount}\left({\text{TA}}_{\text{PRN}},{\text{kg ha}}^{-1}\right)\\ =\text{Aboveground N accumulation of vegetable organ at anthesis}\\ -\text{Aboveground N accumulation of vegetable organ at maturity} \end{array}$$

(5) $$\begin{array}{c} \text{Translocation rate }\left({\text{TR}}_{\text{PRN}},\%\right)\\ =\text{Pre-anthesis N translocation}\\ ÷\text{Aboveground N accumulation at anthesis}\times 100 \end{array}$$

(6) $$\begin{array}{c} \text{Contribution rate of pre-anthesis N translocation to grain N}\\ \left({\text{CR}}_{\text{PRN}},\%\right)\\ =\text{Pre-anthesis N translocation}\\ ÷\text{Grain N accumulation at maturity}\times 100 \end{array}$$

(7) $$\begin{array}{c} \text{Contribution rate of post-anthesis N accumulation to grain }\\ \text{N}({\text{CR}}_{\text{ATM}},\%)\\ \;=\;\left(\text{Post-anthesis N accumulation}\right)\\ ÷\text{Grain N accumulation at maturity}\times 100 \end{array}$$

#### 2.3.3 Grain yield, protein content and protein yield

At the maturity stage, four representative sampling areas, each measuring 2 m by 1.36 m, were randomly selected within each plot. The wheat plants within these areas were manually harvested to assess grain yield. Following harvest, the plants were air−dried, threshed, and the grain obtained was weighed. To accurately determine grain moisture content and dry weight, 100 g samples of the air−dried grain were further oven−dried at 90°C for 30 minutes and then at a reduced temperature of 65°C for a duration of 24 hours. Grain yield calculations for each plot were standardized to a uniform moisture content of 12.5%, using the air−dried grain weight and its determined water content to adjust the figures accordingly. The grain protein content was then calculated by multiplying the grain’s total N content, expressed in g kg^−1^, by the factor 0.57—which is specific to cereal grains. Lastly,the protein yield, expressed in kilograms per hectare (kg ha^−1^), was calculated using the Equation 8, as documented by Huang et al. (2021):


(8) $$\begin{array}{c} \text{Protein yield}\left({\text{kg ha}}^{-1}\right)=0.875\times \text{Grain yield}\times \text{Grain protein content}\\ ÷100,\text{ where }0.875\text{ and }100\text{ were the conversion coefficients}. \end{array}$$

#### 2.3.4 N use efficiency

The N fertilizer partial factor productivity, N internal efficiency, N uptake efficiency and N harvest index were calculated using the Equations 9-12, respectively (Huang et al. 2017):


(9) $$\text{N fertilizer partial factor productivity}(\text{PFPN},{\text{kg kg}}^{-1})={\text{Y}}_{\text{g}}÷{\text{F}}_{\text{N}}$$

(10) $$\text{N uptake efficiency}(\text{NUPE},{\text{kg kg}}^{-1})={\text{NA}}_{\text{M}}÷{\text{F}}_{\text{r}}$$

(11) $$\text{N internal efficiency}(\text{NIE},{\text{kg kg}}^{-1})={\text{Y}}_{\text{g}}÷{\text{NA}}_{\text{M}}$$

(12) $$\text{N harvest index}(\text{NHI},\%)={\text{NA}}_{\text{g}}÷{\text{NA}}_{\text{M}}\times 100$$

where Y_g_ is the grain yield (kg ha^−1^); and F_N_ is the fertilizer rate for N (kg N ha^−1^); NA_M_ is the total N accumulation in aboveground parts at maturity (kg ha^−1^); NA_g_ is the N accumulation in grain at maturity (kg ha^–1^).

### 2.4 Statistical analysis

The means of the data for each treatment were computed by averaging the values across all plots. Differences between these means were assessed using analysis of variance (ANOVA) followed by the Least Significant Difference (LSD) test at a significance level of *P* = 0.05. These statistical analyses were conducted using the SPSS statistical software (version 18, IBM Corp., Chicago, IL, USA). Graphical representations of the data were created with Microsoft Excel 2010.

## 3 Results

### 3.1 Soil nitrate−N

**Figure 3:**
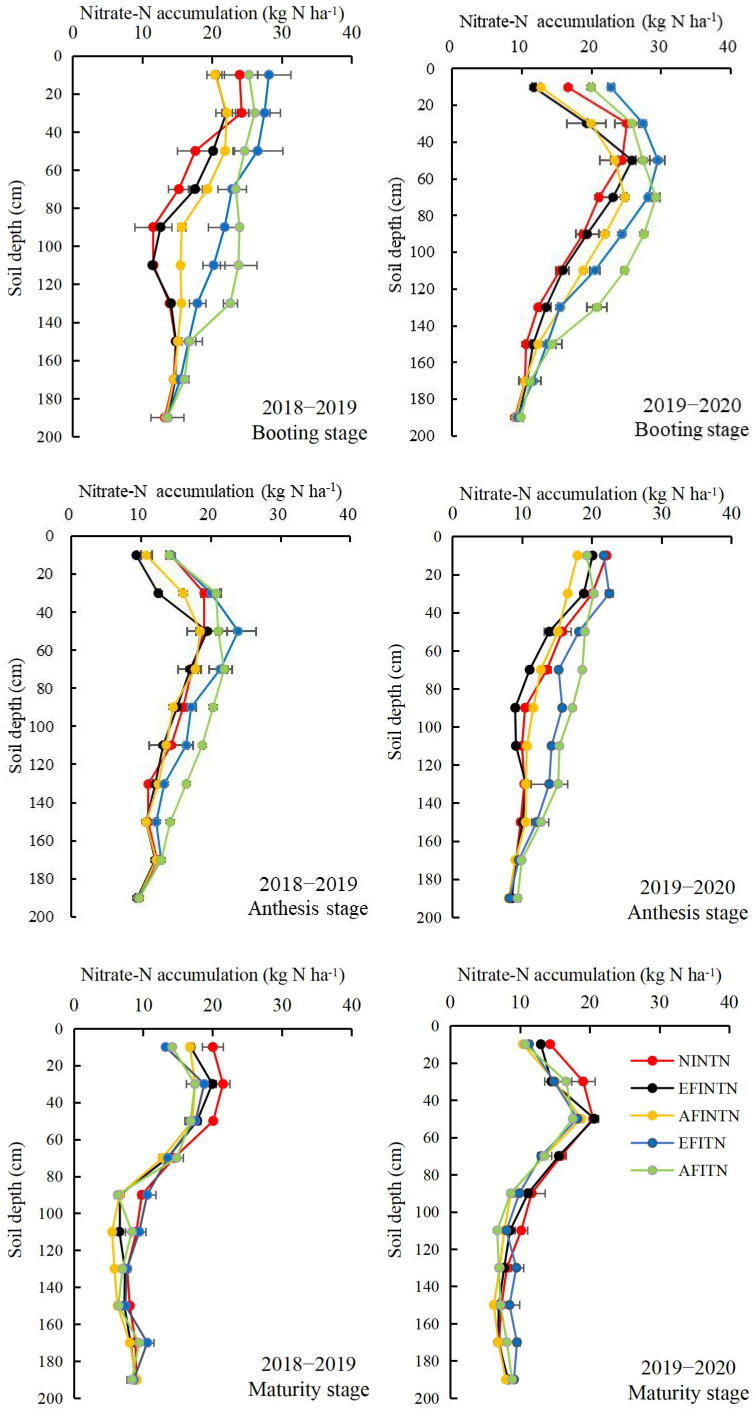
Soil nitrate−N accumulation at the different soil depths affected by the FI, AFI and TN techniques in 2018−2019 and 2019−2020. NINTN, no irrigation with no topdressing N; EFINTN, every furrow irrigation with no topdressing N; AFINTN, alternate furrow irrigation with no topdressing N; EFITN, every furrow irrigation with topdressing N; AFITN, alternate furrow irrigation with topdressing N. Bars indicated standard deviation.

Significant differences in soil nitrate−N accumulation were observed among the treatments at the booting, anthesis, and maturity stages in both years, as shown in **Table 2** and **Figure 3**. Compared to NINTN treatment, the EFINTN treatment did not alter the soil nitrate−N accumulation at the booting stage. However, it resulted in a notable reduction in soil nitrate−N accumulation by 7.0−9.4% at the anthesis stage and 7.3−12.1% at the maturity stage, respectively. These reductions suggest that furrow irrigation at the jointing stage enhanced the uptake of soil nitrate−N by the wheat crop. In contrast, when compared to the every furrow irrigation (EFI) treatments, the alternate furrow irrigation (AFI) treatments showed increased soil nitrate−N accumulation levels at booting and anthesis stages, with a marked decrease in the upper soil layer but a significant increase in the 80−140 cm soil layer. Nevertheless, at the maturity stage, AFI treatments exhibited a significant reduction in soil nitrate−N accumulation, ranging from 6.8−8.1%. Irrespective of the irrigation method, the topdressing nitrogen (TN) treatments exhibited a significant increase in soil nitrate−N accumulation at booting (28.3%) and anthesis (24.6%) stages when compared to the no topdressing nitrogen (NTN) treatments. However, the TN treatments did not show a significant effect on the soil nitrate−N accumulation at maturity. The findings suggest that employing both AFI and TN techniques at the jointing stage could enhance soil N availability during the middle growth stages of winter wheat but had no beneficial impact at maturity. Across the two years, the soil nitrate−N accumulation at the maturity stage was observed to be AFITN = AFINTN< EFITN = EFINNT< NINTN and the AFITN, AFINTN, EFITN, and EFINNT treatments reduced the soil nitrate−N accumulation by 9.7%, 17.1%, 8.5%, and 14.6%, respectively, compared to the NINTN treatment. These results indicated that implementing the AFINTN technique effectively decreased the risk of nitrate−N accumulation, even with an application of 60 kg N ha^−1^ at the jointing stage.

**Table 2:** Soil nitrate−N accumulation kg N ha^-1^) at booting, anthesis and maturity stages affected by the FI, AFI and TN techniques in 2018−2019 and 2019−2020.

Treatment	Booting stage		Anthesis stage		Maturity stage	
	2018−2019	2019−2020	2018−2019	2019−2020	2018−2019	2019−2020
NINTN	160.5 c	163.8 cd	144.0 c	129.3 c	128.6 a	122.2 a
EFINTN	161.5 c	160.4 d	130.5 e	120.3 d	113.1 bc	113.3 b
AFINTN	173.5 b	169.0 c	136.0 d	122.7 d	105.5 d	102.3 c
EFITN	210.6 a	202.7 b	161.2 b	151.2 b	117.4 b	112.1 b
AFITN	216.2 a	210.6 a	169.9 a	157.0 a	109.3 cd	104.9 c
*F*−value						
Year (Y)	4.0ns		8.2*		23.7**	
Treatment(T)	201.3**		322.5**		82.3**	
Y*T	1.5ns		0.7ns		2.7ns	

NINTN, no irrigation with no topdressing N; EFINTN, every furrow irrigation with no topdressing N; AFINTN, alternate furrow irrigation with no topdressing N; EFITN, every furrow irrigation with topdressing N; AFITN, alternate furrow irrigation with topdressing N. Means in a column followed by the different lowercase letters within a year are significantly different at P<0.05. The symbol *, ** and ns indicated that the P are< 0.05,< 0.01 and > 0.05.

### 3.2 Plant N accumulation, translocation, and allocation

#### 3.2.1 Periodical N accumulation during various growth stages and its rate to aboveground N at maturity

Aboveground N accumulation during different growth stages, as well as its proportion of aboveground total N accumulation at maturity, were influenced by the furrow irrigation (FI), AFI, and TN techniques over the two years, as detailed in **Table 3**. Compared to the NINTN treatment, the EFINTN treatment significantly boosted both the overall N accumulation, specifically the N accumulation from jointing to anthesis and from anthesis to maturity. Consequentially, the N accumulation at anthesis was increased by 14.8% and the aboveground N accumulation at maturity by 22.8%. Compared to the EFI treatments, AFI treatments under the same TN rates decreased both the N accumulation from jointing to anthesis and its rate to aboveground N accumulation at maturity. Nevertheless, they significantly raised N accumulation from anthesis to maturity by 63.2% and its rate to aboveground total N accumulation at maturity by 48.9%, culminating in a 9.4−10.1% increase in aboveground N accumulation at maturity across the two years. Additionally, within the same FI technique, TN treatments markedly raised the N accumulation from jointing to anthesis by 42.9−50.0% and the rate of periodical N accumulation from jointing to anthesis to aboveground N accumulation at maturity by 12.9−18.0%, as well as the N accumulation from anthesis to maturity by 63.5−97.9% and the rate of periodical N accumulation from anthesis to maturity to aboveground N accumulation at maturity by 29.5−56.1%, ultimately enhancing the aboveground N accumulation at maturity by 23.1−29.5%, averaged across the two years. These findings demonstrate that both AFI and TN techniques have the potential to promote aboveground N accumulation during the mid and late growth periods and its rate to aboveground total N accumulation at maturity in dryland winter wheat.

**Table 3:** Periodical N accumulation amount and its rate to aboveground N accumulation at maturity affected by the FI, AFI and TN techniques in 2018−2019 and 2019−2020.

Year	Treatment	NA_JTA_ (kg ha^−1^)	NA_A_ (kg ha^−1^)	NA_ATM_ (kg ha^−1^)	NA_M_ (kg ha^−1^)	R_JTA_ (%)	R_ATM_ (%)
2018−2019	NINTN	33.1 d	127.1 d	18.3 d	145.3 e	22.8 b	12.5 d
2018−2019	EFINTN	49.9 bc	143.8 bc	29.6 c	173.4 d	28.8 a	17.1 c
2018−2019	AFINTN	45.0 c	139.0 c	51.0 b	190.0 c	23.7 b	26.9 b
2018−2019	EFITN	63.5 a	157.4 a	56.5 b	213.8 b	29.7 a	26.4 b
2018−2019	AFITN	53.6 b	147.5 b	86.3 a	233.8 a	22.9 b	36.9 a
2019−2020	NINTN	17.6 c	117.2 c	8.6 d	125.8 e	13.9 c	6.9 d
2019−2020	EFINTN	37.2 b	136.7 b	24.5 c	161.2 d	23.0 b	15.2 c
2019−2020	AFINTN	32.4 b	132.0 b	45.4 b	177.4 c	18.3 c	25.6 b
2019−2020	EFITN	59.0 a	158.5 a	50.2 b	208.7 b	28.2 a	24.0 b
2019−2020	AFITN	58.6 a	158.1 a	71.6 a	229.8 a	25.5 ab	31.2 a
*F*−value							
Year (Y)		29.0**	3.7ns	28.6**	3.7ns	16.1**	20.4**
Treatment(T)		72.4**	100.4**	214.5**	100.4**	15.5**	129.6**
Y*T		6.3**	8.7**	1.4ns	8.7**	4.3*	1.7 ns

NINTN, no irrigation with no topdressing N; EFINTN, every furrow irrigation with no topdressing N; AFINTN, alternate furrow irrigation with no topdressing N; EFITN, every furrow irrigation with topdressing N; AFITN, alternate furrow irrigation with topdressing N. NAJTA, the aboveground N accumulation from jointing to anthesis; NAA, the aboveground N accumulation at anthesis; NAATM, the aboveground N accumulation from anthesis to maturity; NAM, the aboveground N accumulation at maturity; RJTA, the rate of NAJTA to NAM; RATM, the rate of NAATM to NAM. Means in a column followed by the different lowercase letters within a year are significantly different at P < 0.05. The symbol *, ** and ns indicated that the P are < 0.05, < 0.01 and > 0.05.

#### 3.2.2 Pre-anthesis N translocation and its contribution to grain

**Table 4** illustrates the translocation amount and translocation rate of pre-anthesis N from vegetative organs to grains, which can be ranked as follows: the TN treatments exhibited higher values than NTN treatments, and the EFI treatments showed higher values than AFI treatments. Conversely, the contribution rates of pre-anthesis N to grain N were higher for the NTN treatments than TN treatments, and for the AFI treatments than EFI treatments. Consequently, the optimized values for pre-anthesis N translocation amount and its rate were achieved under the EFITN treatment. However, the contribution rates of pre-anthesis N translocation to grain N was most substantially recorded under the NINTN treatment over the two years.

**Table 4:** N translocation from vegetative organ to grain, and contribution rate of post-anthesis N accumulation to grain of winter wheat affected by the FI, AFI and TN techniques in 2018−2019 and 2019−2020.

Year	Treatment	TA_PRN_ (kg ha^−1^)	TR_PRN_ (%)	CR_PRN_ (%)	CR_ATM_ (%)
2018−2019	NINTN	91.9 b	72.3 a	83.5 a	16.5 d
2018−2019	EFINTN	99.7 a	69.3 b	77.1 b	22.9 c
2018−2019	AFINTN	92.8 b	66.8 bc	64.5 c	35.5 b
2018−2019	EFITN	104.2 a	66.2 c	64.9 c	35.1 b
2018−2019	AFITN	91.1 b	61.8 d	51.3 d	48.7 a
2019−2020	NINTN	73.6 c	62.7 a	89.5 a	10.5 d
2019−2020	EFINTN	84.0 b	61.5 a	77.4 b	22.6 c
2019−2020	AFINTN	77.0 c	58.3 b	62.9 c	37.1 b
2019−2020	EFITN	95.8 a	60.4 ab	65.6 c	34.4 b
2019−2020	AFITN	92.4 a	58.4 b	56.4 d	43.6 a
*F*−value					
Year (Y)		60.8**	168.2**	3.9ns	3.9ns
Treatment(T)		17.1**	21.9**	118.6**	118.6**
Y*T		6.0**	4.1*	1.9ns	1.9ns

NINTN, no irrigation with no topdressing N; EFINTN, every furrow irrigation with no topdressing N; AFINTN, alternate furrow irrigation with no topdressing N; EFITN, every furrow irrigation with topdressing N; AFITN, alternate furrow irrigation with topdressing N. TA_PRN_, the translocation amount of pre-anthesis N; TR_PRN_, the translocation rate of pre-anthesis N; CR_PRN_, the contribution rates of pre-anthesis N translocation to grain N; CR_ATM_, the contribution rate of NA_ATM_ to grain N. Means in a column followed by the different lowercase letters within a year are significantly different at P< 0.05. The symbol *, ** and ns indicated that the P are< 0.05,< 0.01 and > 0.05.

Both AFI and TN treatments, when compared within the same TN rate or FI technique, significantly enhanced the contribution rate of aboveground N accumulation from anthesis to maturity to grain N. The highest values for aboveground N accumulation from anthesis to maturity to grain N were observed under the AFITN treatment, followed by EFITN or AFINTN, then enhanced compared to EFINTN, and, finally, NINTN. This descending order of effectiveness was consistent in the two years. Notably, the differences in aboveground N accumulation from anthesis to maturity to grain N among treatments were statistically significant, with the exception of no significant difference discerned between EFITN and AFINTN.

#### 3.2.3 N allocation in various organs at maturity

**Table 5** presents data indicating that the EFINTN treatment significantly increased N allocation in different winter wheat organs in both years when compared with the NINTN treatment, with the exception of the glume + rachis in 2018−2019. Furthermore, when compared to the EFI treatments, the AFI treatments boosted grain N allocation by 11.6% averaged across the years and TN techniques. In comparison to the NTN treatments, the TN treatments significantly increased N allocation: 28.5% in grain, 21.2% in the glume + rachis, and 19.8% in the stem + leaf + sheath, averaged across the years and FI techniques. Finally, the AFITN treatment yielded the greatest N allocation in grain, which increased by 80.2%, 44.1%, 28.6%, and 11.4% when compared to the NINTN, EFINTN, AFINTN, and EFITN respectively, averaged across the two years. These findings underscore that N allocation in various organs of winter wheat at maturity is significantly influenced by both FI and AFI, as well as TN techniques. Moreover, the data suggest that the synergistic interaction between AFI and TN is a major determinant of N distribution patterns in winter wheat.

**Table 5:** N allocation (kg ha^−1^) in various organs of wheat at maturity affected by the FI, AFI and TN techniques in 2018−2019 and 2019−2020.

Treatment	Grain		Glume + rachis		Stem + leaf + sheath	
	2018−2019	2019−2020	2018−2019	2019−2020	2018−2019	2019−2020
NINTN	110.2 e	82.2 e	13.3 c	13.1 c	21.8 d	30.5 d
EFINTN	129.3 d	108.6 d	13.7 c	15.5 b	30.5 c	37.2 c
AFINTN	143.9 c	122.4 c	13.3 c	15.6 b	32.8 b	39.4 c
EFITN	160.6 b	145.9 b	16.0 b	18.4 a	37.2 a	44.3 b
AFITN	177.4 a	164.0 a	17.5 a	18.5 a	38.9 a	47.2 a
*F*−value						
Year (Y)	259.9**		69.7**		226.9**	
Treatment(T)	454.3**		106.8**		141.0**	
Y*T	4.6**		7.1**		0.7ns	

NINTN, no irrigation with no topdressing N; EFINTN, every furrow irrigation with no topdressing N; AFINTN, alternate furrow irrigation with no topdressing N; EFITN, every furrow irrigation with topdressing N; AFITN, alternate furrow irrigation with topdressing N. Means in a column followed by the different lowercase letters within a year are significantly different at P< 0.05. *Significant at P< 0.05; ** significant at P< 0.01; ns, not significant.

### 3.3 Grain yield, protein content, and protein yield

**Table 6** reveals significant variances among treatments in terms of grain yield, protein content, and protein yield. The AFITN treatment achieved the highest grain and protein yields, followed by EFITN, AFINTN, EFINTN, and lastly, NINTN, all in descending order with statistical significance (*P*< 0.05) over the two years. Regarding grain protein content, there was no statistical difference was detected between EFINTN and AFINTN treatments; but there was a respective decrease of 8.8% and 10.7% when compared to the NINTN treatment, averaged across the two seasons. TN treatments under same furrow irrigation (FI) technique exhibited significant increases in grain protein content—ranging from 4.4% to 14.9%—when compared to the NTN treatments. Of note, the enhancements under AFI were higher than those under EFI. Furthermore, in comparison to the NINTN treatment, the grain protein content under the EFITN treatment diminished significantly by 2.7% to 4.1%, whereas it did not decrease under the AFITN treatment and in fact exhibited an increase of 2.7% in 2019−2020. These results conclusively indicates that the strategic coupled application of AFI and TN techqiue at the jointing stage can substantially enhance grain yield and either sustain or potentially increase the protein content, thereby significantly elevating the protein yield of dryland winter wheat.

**Table 6:** Grain yield, protein content, protein yield of winter wheat affected by the FI, AFI and TN techniques in 2018−2019 and 2019−2020.

Treatment	Grain yield (kg ha^–1^)		Protein content (%)		Protein yield (kg ha^−1^)	
	2018−2019	2019−2020	2018−2019	2019−2020	2018−2019	2019−2020
NINTN	4853 e	3571 e	14.8 a	15.0 b	628.1 e	468.6 e
EFINTN	6202 d	5209 d	13.6 c	13.4 d	736.9 d	618.9 d
AFINTN	6999 c	5953 c	13.4 c	13.4 d	820.1 c	697.8 c
EFITN	7392 b	6515 b	14.2 b	14.6 c	915.6 b	831.8 b
AFITN	7918 a	6943 a	14.6 a	15.4 a	1011.2 a	934.8 a
F−value						
Year (Y)	625.5**		13.3**		260.0**	
Treatment(T)	759.7**		94.1**		454.4**	
Y*T	3.1*		5.7**		4.6**	

NINTN, no irrigation with no topdressing N; EFINTN, every furrow irrigation with no topdressing N; AFINTN, alternate furrow irrigation with no topdressing N; EFITN, every furrow irrigation with topdressing N; AFITN, alternate furrow irrigation with topdressing N. Means in a column followed by the different lowercase letters within a year are significantly different at P< 0.05. The symbol * and ** indicated that the P are< 0.05,< 0.01 and > 0.05.

### 3.4 N use efficiency

Across both years, a significant divergence in N fertilizer partial factor productivity (PFPN), N uptake efficiency (NUPE), and N internal efficiency (NIE) was observed among the different treatments, generally ordered as AFINTN > EFINTN > AFITN > EFITN > NINTN (**Table 7**). Specifically, the PFPN under the AFINTN treatment surpassed EFINTN, AFITN, EFITN, and NINTN by 13.4%, 17.3%, 25.6%, and 53.9%, respectively, as well as NUPE by 9.8%, 6.5%, 17.0%, and 35.7%, while NIE showed improvements of 3.4%, 9.8%, 7.0%, and 13.9%, averaged across the two years. These results suggest that both FI and AFI techniques bolster N use efficiency, whereas the TN technique had a diminishing effect. In 2018–2019 year, the differences in the N harvest index (NHI) among treatments were not statistically significant. Nonetheless, in 2019–2020, the NHI was ranked as AFITN > EFITN > AFINTN > EFINTN > NINTN, with significant differences among all treatments exception between EFITN and AFINTN or AFITN.

**Table 7:** N use efficiency of winter wheat affected by the FI, AFI and TN techniques in 2018−2019 and 2019−2020.

Treatment	PFPN (kg kg^−1^)		NUPE (kg kg^−1^)		NIE (kg kg^−1^)		NHI (%)	
	2018−2019	2019−2020	2018−2019	2019−2020	2018−2019	2019−2020	2018−2019	2019−2020
NINTN	28.1 e	20.7 e	0.84 d	0.73 e	33.4 d	28.4 e	75.8 a	65.3 d
EFINTN	36.0 b	30.2 b	1.01 b	0.93 c	35.8 b	32.3 b	74.6 a	67.3 c
AFINTN	40.6 a	34.5 a	1.10 a	1.03 a	36.8 a	33.6 a	75.7 a	69.0 b
EFITN	31.8 d	28.0 d	0.92 c	0.90 d	34.6 c	31.2 c	75.1 a	69.9 ab
AFITN	34.1 c	29.9 c	1.01 b	0.99 b	33.9 cd	30.2 d	75.9 a	71.4 a
F−value								
Year (Y)	642.0**		88.1**		379.0**		374.4**	
Treatment(T)	398.3**		224.8**		60.9**		10.0**	
Y*T	9.5**		8.3**		2.8ns		8.7**	

NINTN, no irrigation with no topdressing N; EFINTN, every furrow irrigation with no topdressing N; AFINTN, alternate furrow irrigation with no topdressing N; EFITN, every furrow irrigation with topdressing N; AFITN, alternate furrow irrigation with topdressing N. PFPN: N fertilizer partial factor productively; NUPE: N uptake efficiency, NIE: N internal efficiency; NHI: N harvest index. Means in a column followed by the different lowercase letters within a year are significantly different at P<0.05. The symbol * and ** indicated that the P are< 0.05,< 0.01 and > 0.05.

## 4 Discussion

### 4.1 Impact of FI, AFI, and TN at jointing on soil nitrate−N

Soil nitrate−N, the predominant form of N absorbed by crops in dry farming regions, is influenced by a myriad of factors including N fertilizer application, plant uptake and utilization, soil water penetration, and leaching processes (Zhou and Buterbach-Bahl, 2014; Wang and Li, 2019). Our study found that compared to the NINTN treatment, the EFINTN treatment resulted in lower soil nitrate−N accumulation at both the anthesis and maturity stages, suggesting that furrow irrigation (FI) reduced soil nitrate−N accumulation in dryland winter wheat farming systems. This reduction was primarily attributed to the improved N uptake by the crop due to better soil moisture from irrigation (**Table 2**, Adel et al., 2019). When comparing irrigation methods, alternate furrow irrigation (AFI) elevated soil nitrate−N accumulation at the booting and anthesis stages, particularly within the 80−140 cm soil layer, compared to every furrow irrigation (EFI). This enhancement, however, diminished progressively with wheat growth, especially from anthesis to maturity, leading to lower soil nitrate−N accumulation under AFI than under EFI at the maturity stage (**Table 2**, **Figure 3**). These findings suggest that AFI enhances subsoil nitrate−N accumulation in the mid−growth stages but reduces it at maturity stage of wheat, potentially due to the dual role of water movement and plant N uptake. Firstly, soil nitrate−N is prone to download leaching with irrigation water, more irrigation amount in the irrigated furrow resulted in less nitrate−N in the upper and more nitrate−N in the deeper soil layers (Khan et al., 2020). Secondly, AFI’s moist environment is known to improve plant N requirement (Jia et al., 2021) and the distribution, density, and activity of crop root (Liang et al., 2000; Wang et al., 2014), all of these would help to increase soil nitrate-N absorption by wheat (Jia et al., 2021).

Previous research demonstrated that N fertilizer topdressing can modulate soil nitrate−N accumulation, and its effects influenced by water management strategies (Zhao and Yu, 2006; Shi et al., 2012; Li et al., 2020). Han et al. (2014) compared AFI and N topdressing paired to conventional irrigation and fertilization, noting that the former increased nitrate−N in the upper soil (0–60 cm) by 30−60% while reducing it in the deeper layers (60–200 cm) by 8−44% in summer maize farmland, mainly due to the enhancement of N requirement and root morphology from the optimized moisture introduced by AFI. In our investigation, TN treatments with 60 kg N ha^−1^ topdressing at the jointing stage consistently improved the soil nitrate−N accumulation in most soil layers at the booting and anthesis stages, regardless of the irrigation techqiue employed, although the differences waned as the wheat matured. Notably, the combination of AFI and TN synergistically increased the soil nitrate−N accumulation, leading to the AFITN treatment exhibiting the highest soil nitrate−N accumulation during the booting and anthesis stages (**Table 2**, **Figure 3**). This implies that integrating AFI with TN at the jointing stage can bolster the soil nitrate−N accumulation and providing a favorable N supply in wheat’s middle growth phase. This soil nitrate−N accumulation enhancement mainly ascribed the N from topdressing and soil N mineralization. The greater moisture in the irrigated furrow and the partially dry soils under AFI system has strong air permeability, which generate favorable conditions for microbial activity, thus enhancing soil N mineralization and affecting the nitrate-N distribution in soil (Tian et al., 2016; Pu et al., 2022).

In terms of environmental risk, less residual nitrate−N at wheat maturity is preferable for reducing N loss and water pollution (Huang et al., 2017; Lu et al., 2019). Researches have indicated that FI technique can contribute significantly to nitrate−N leaching and groundwater contamination within agricultural systems, potentially leaching up to 40% of the nitrate−N in the root development zone (Siyal et al., 2012; Iqbal et al., 2019). Luo et al. (2020) observed that supplemental irrigation under rainfed systems could substantially increase soil nitrate−N concentration. However, studies like those by Skinner et al. (1999) have shown that AFI may lessen the risk of nitrate−N leaching when irrigation water is weekly applied, similar to EFI techniques throughout crop growth stages. Optimal irrigation and fertilization under furrow irrigation system shown by Adel et al. (2019) was also found to increase root water and N uptake while reducing nitrate−N losses, compared to conventional practices. Variations in experimental conditions may account for differences in study outcomes. In our case, FI treatments exhibited a substantial reduction in the soil nitrate−N accumulation at the maturity stage of wheat, especially under AFI treatments, meaning that AFI at jointing is prone to reduce the soil nitrate−N accumulation at maturity in wheat cropping system (**Table 2**, **Figure 3**). This aligns with Jia et al. (2021), who found that AFI with 180 mm of water reduced nitrate−N leaching by an average of 8.3% and 16.6% compared to flood irrigation with 180 mm and 270 mm, respectively. In turn, TN treatments did not show a discernible effect on the soil nitrate−N accumulation at maturity stage of winter wheat (**Table 2**, **Figure 3**). This showed that the 60 kg N ha^-1^ topdressing under one-off furrow irrigation did not increase the environment risk from nitrate−N. Moreover, all combinations of FI and TN techniques decreased soil nitrate−N accumulation at the maturity stage of winter wheat when compared to NINTN, particularly highlighting the effectiveness of AFITN in minimizing the risk of nitrate−N leaching (**Table 2**, **Figure 3**). These results indicated that at the maturity stage of winter wheat, the effectiveness of TN-introduced soil nitrate−N accumulation increase was lower than the FI-introduced soil nitrate−N accumulation decrease, leading to a marked decrease of soil nitrate−N accumulation, and thus alleviating the environmental risk.

### 4.2 Influence of FI, AFI and TN at jointing on winter wheat grain yield and protein content

Boosting grain yield remains a primary driver in wheat production, particularly in dryland regions where the potential for yield increases eclipses that of more infertile areas (Wang and Li, 2019). Our research reveals that the techniques of furrow irrigation (FI), alternative furrow irrigation (AFI), and topdressing nitrogen (TN) all contributed to a significant upsurge in winter wheat’s grain yield. This mainly ascribe to the improvement of soil moisture (Wu et al., 2023) and N supply (**Table 2**, **Figure 3**). Notably, the AFITN treatment outperformed the NINTN by 63.2–94.4% in the two years, indicating a substantial margin for yield enhancement in dryland furrow−seeded (FS) wheat systems provided by a coupled application of one-off AFI and TN at the jointing stage.

Evolving living standards have ramped up demand for high−quality grain and wheat flour (Lin et al., 2015). Grain protein content is a critical quality parameter for winter wheat, vital for both food and non−food applications (Luo et al., 2018; Hu et al., 2021). In our study, compared to the NINTN treatment, the FI technique under NTN (EFINTN and AFINTN) treatments decreased grain protein content by 8.1–10.6%. This reduction is often linked to the dilutive effect on N concentration due to the increased grain yield (Sissons et al., 2014; Hu et al., 2021), as supported by Luo et al. (2020), who documented a 5.4% drop in protein content in FI−treated winter wheat. Similarly, Sarker et al. (2020) found that AFI maintained yield and grain N content in maize despite a 37.0% reduction in irrigation water in a sub−tropical South Asian climate.

The interplay of plant N requirement, accumulation, translocation, and allocation, and the N supply-demand relationships in various wheat growth stages greatly dictates variations in wheat yield and grain protein content, all of which can be refined through judicious irrigation and N management (Aziz et al., 2018; Jia et al., 2021; Damme et al., 2022). An Iranian field experiment illustrated AFI’s superiority over EFI in bolstering shoot N uptake and grain protein content in the FS barley system (Ghasemi-Aghbolaghi and Sepaskhah, 2018). Similarly, in China’s Huang−Huai−Hai Plain, AFI distributed at wintering and grain filling stage outpaced EFI treatments at anthesis stage with the same irrigation amount, improving both grain yield and grain N accumulation (Jia et al., 2021). Our investigation further corroborates that, AFI did not significantly alter wheat grain protein content under NTN while increased by 2.8–5.5% under TN (**Table 6**), indicating the TN treatments could minimized N dilution under AFI system despite the augmented yield. The reason being, although AFI did not noticeably alter the N accumulation at anthesis and the translocation amount of pre-anthesis N, it considerably enhanced the N accumulation from anthesis to maturity, and its contribution to grain N in winter wheat, supporting the significant increase of grain N accumulation at maturity (**Table 4**). Four potential reasons are discernible: (1) AFITN’s promotion of robust root systems (Wang et al., 2012) and supply-demand status (Zhang et al., 2012), leading to heightened N uptake and assimilation (Jia et al., 2021; Yang et al., 2021); (2) the improved soil moisture and soil nitrate−N accumulation enhancing the N accumulation from anthesis to maturity and its contribution to grain N, resulting in the enhancement of grain protein content (**Table 6**); (3) the 2−folds irrigation volume within the irrigated furrow under AFI allowing the deep soil deposition of fertilizer N, which eluded fertilizer N loss via volatilization, favored root development and N uptake during later growth phases (Wang et al., 2014). (4) the moderate moisture levels under AFI assist N cycle phases, improving N availability and leading to amplified grain N storage (Ashraf et al., 2016).

Optimized N supply at key growth stages is often used to improve plant N characteristics, increase crop yield and grain protein content (Aziz et al., 2018; Damme et al., 2022). Wang et al. (2021) found that employing topdressing N technique at the jointing stage significantly or extremely significantly enhanced the N accumulation, yield, protein content of all the tested wheat. Similar results were also obtained in this study, employed the 60 kg N kg^-1^ topdressing at the jointing stage show significant advantages in N accumulation at anthesis, N transloctaion amout of pre-anthesis N, N accumulation from anthesis to maturity and its contribution to grain N (**Tables 3**, **4**), grain N allocation (**Table 5**) and consequently boosting grain yield and protein content (**Table 6**), thereby addressing the grain protein content reduction caused by FI and AFI. As a result, the AFITN combination yielded top levels in grain yield, and protein content, culminating in an optimal protein yield. These results illustrated that, the topdressing N may employ to balance the N supply-demand for simultaneously increasing grain protein quality and the FI-introduced increase of N requirement.

### 4.3 Enhancement of N use efficiency by FI, AFI, and TN at jointing

Enhancing N use efficiency (NUE) is crucial for sustainable and environmentally friendly wheat production (Zhang et al., 2020; Zhao et al., 2023b). Studies, such as Jia et al. (2021), found that alternate furrow irrigation (AFI), with an equivalent water volume of 150 mm, significantly increased winter wheat’s N uptake efficiency (NUPE) by 8.0% compared to the flood irrigation. Han et al. (2014) also demonstrated that AFI combined with separated N fertilizer applications, improved N agronomic efficiency by 36−56% in summer maize compared to conventional irrigation methods. Comparable enhancements in N use efficiency (NUE) of AFI under the same irrigation quantities were recorded with sugar beets in Turkey (Mon et al., 2016) and safflower in Iran (Shahrokhnia and Sepaskhah, 2016). Conducted under semi−humid, drought−prone conditions, our study shows that implementing furrow irrigation (FI) with 75 mm at the jointing stage significantly enhances the partial factor productivity of nitrogen (PFPN), NUPE, and N internal efficiency (NIE) by 24.2−55.6%, 16.4−36.0%, and 3.9−14.2% respectively across the two years. Herein, the gains under the AFI treatments surpassed those under the EFI treatments, whereas improvements under the TN treatments fell short of the NTN treatments. These outcomes suggest that NUE in winter wheat can be markedly augmented through FI and AFI, particularly for AFI under both NTN and TN conditions. The enhancement results chiefly from FI and AFI’s significant increase in soil moisture content (Wu et al., 2023) and the optimized nitrate−N distribution from booting to maturity (**Table 2**, **Figure 3**), thus balancing the plant N requirement and soil N supply, finally elevating the plant N absorption, accumulation, and utilization proficiency (Chen et al., 2020; Jia et al., 2021). Contrastingly, there have been instances where AFI was associated with reduced NUE in winter wheat (Sepaskhah and Hosseini, 2008) and plant N uptake for sugar beets in Iran (Mehdi et al., 2017). Ghasemi-Aghbolaghi and Sepaskhah (2018) also reported a decrease in both N internal efficiency (NIE) and NAE for barley using AFI in Iran. Variations in experimental conditions such as climate, soil properties, and cultivation managements may account for differences in study outcomes. In this study, although the precipitation amount in whole year and wheat growing season is higher, the N uptake efficiency in 2019−2020 was obviously lower than 2018−2019, which mainly because the consecutively extreme high temperature (from2 May to 4 May, over 40°C) occurred during early grain filling stage in 2020−2021, thus resulting in a marked decrease in grains per spike and 1000-grain weight (Wu et al., 2023), and finally decreasing the grain yield (**Table 6**) and grain N accumulation (**Table 5**). Ru et al. (2022) also reported that short-term extreme heat stress after anthesis resulted in a pronounced decrease in yield and NUE by reducing grain number per spike and thousand kernel weight. Hence, the effects of climate such as temperature should be considered in wheat production to enhance wheat yield and nutrient use efficiency.

N management remains a pivotal influence on N use efficiency in winter wheat, and the effectiveness relates to water conditions (Ji et al., 2021; Zain et al., 2021; Zhao et al., 2023b). In our study, compared to NTN, TN treatments of 60 kg ha^−1^ topdressing at the jointing stage has a dampening impact on PFPN, NUE, and NIE under both EFI and AFI, but these efficiencies still register significantly higher than that under the traditional NINTN treatment. This maybe ascribe to FI’s promotion of N uptake and assimilation (Jia et al., 2021; Yang et al., 2021). In our case, the decrease of soil nitrate−N accumulation (**Figure 3**, **Table 2**) at different growth stages among treatments can also provide an evidence for the marked difference of soil nitrate−N uptake by wheat This reveals that the potential negative influence of TN on N use efficiency can be mitigated by FI and AFI strategies, especially in dryland where at least one-off irrigation is assured.

## 5 Conclusion

The obtained results indicate that under the furrow seeding system, when implemented the furrow irrigation (FI), alternate furrow irrigation (AFI), and topdressing nitrogen (TN) techniques, significantly enhances nitrate−N levels at the booting and anthesis stages. This elevation leads to increased aboveground N accumulation, particularly post-anthesis, which contributes considerably to grain yield and protein yield. Concurrently, TN treatment notably enhances grain protein content, while AFI markedly improves N use efficiency. Therefore, the coupled application of AFI and TN not only boosts yield, quality, and efficiency but also mitigates soil nitrate−N residues at the maturity stage of winter wheat production. This coupled strategy holds great promise for dryland regions where a one−off irrigation event is assured during the growth stages of winter wheat.

## Data availability statement

The raw data supporting the conclusions of this article will be made available by the authors, without undue reservation.

## Author contributions

MH: Data curation, Formal analysis, Investigation, Writing – original draft, Writing – review & editing, Software. WL: Data curation, Formal analysis, Writing – review & editing. CH: Data curation, Formal analysis, Writing – review & editing. JW: Conceptualization, Investigation, Project administration, Writing – review & editing. HW: Conceptualization, Investigation, Project administration, Writing – review & editing. GF: Conceptualization, Project administration, Writing – review & editing. MS: Writing – review & editing. YL: Conceptualization, Funding acquisition, Project administration, Writing – review & editing, Investigation. GL: Conceptualization, Funding acquisition, Project administration, Software, Writing – review & editing.
